# The blame game of the US baby formula crisis

**DOI:** 10.1002/puh2.36

**Published:** 2022-11-29

**Authors:** Lila K. Chamlagai, Elizabeth Silva, Anna Alikhani

**Affiliations:** ^1^ Department of Behavioral Health and Social Science Brown University School of Public Health Providence Rhode Island USA

## Abstract

The United States cannot meet the demand for infant formula. The nation is currently experiencing a problem due to the industry being controlled by a small number of enterprises, states relying on a single supplier, and a ban on importing infant formula from other nations. The four major corporations’ control 89% of the domestic infant formula market creating an industry monopoly within the United States. Second, the dependence of separate states on a single baby formula provider is the cause of the infant formula shortage. In exchange for granting monopolies to formula producers inside specific areas, the federal government is given price cuts on baby formula goods, enabling it to pay for the acquisition. Finally, the United States's inability to import infant formula has significantly exacerbated the current scarcity. However, in the meanwhile, President Joe Biden's administration has put stopgap measures in place to end this shortage, like loosening limits on imports from abroad, but these measures are unlikely to provide long‐term remedies. A multi‐level strategy with long‐lasting structural adjustments is required to avert future infant formula shortages in the United States, but for the time being, caregivers must find individual‐level solutions to meet their children's nutritional needs.

## BABY FORMULA SHORTAGES AREN'T A “NEW” PROBLEM

Currently, the United States is struggling to meet current demands for baby formula [1]. The Biden administration has implemented stopgap strategies to curb this shortage, such as through easing restrictions on imports, but these strategies are unlikely to offer long‐term solutions. While US shortages are alarming, this is not a novel problem as baby formula shortages are pervasive across the globe [2]. During the same year, Australia and New Zealand experienced recalls of infant formula, resulting in supply chain disruptions and leaving caregivers scrambling to find the baby formula [2]. Similarly, several areas within Asia, including China and Hong Kong, experienced an increased demand for breast milk substitutes after recalls impacted their infant formula supply [2]. While these documented infant formula shortages in high‐income countries are important to note, the severity of shortages in low‐ and middle‐income countries (LMICs) is of greater concern due to LMICs’ limited access to clean water, suboptimal infant feeding practices, and the potential for increased natural disasters [3].

Given the numerous shortages and accompanying nutritional concerns noted in high‐income and LMICs alike, US legislation could benefit from considering these commonalities when developing their longer‐term solutions [2, 3]. However, it is also critical to examine the unique political and financial US policies that have contributed to this infant formula crisis. The most noteworthy drivers of shortages are industry monopolies, US states’ reliance on single suppliers, and the absence of infant formula imports [1].

## THE “BIG FOUR” INDUSTRY MONOPOLY

The four major companies control 89% of the domestic infant formula market in the United States [4]. This monopoly exacerbates the felt effects caused by production disruptions in formula products, especially given that some of these companies rely on the same sources for ingredients [4]. For instance, the temporary closure and recall of products at one company's formula plant in Michigan has been cited as an immediate cause of the US formula crisis in conjunction with pandemic‐related supply chain disruptions [5]. Additionally, the monopolization of this market is further perpetuated by the federal government, as it is the biggest customer of infant formula via its Supplemental Nutrition Program for Women, Infants and Children (WIC) [1]. This monopolized system might be established at the federal level, but its disastrous effects will continue to extend to the state level until a more sustainable system for infant formula production is implemented.

## EXCLUSIVE CONTRACTS WITH STATE GOVERNMENTS

Secondly, the infant formula shortage is driven by individual states’ reliance on a single supplier for baby formula [1]. To afford the purchase, the federal government is granted price breaks on baby formula products in exchange for awarding monopolies to formula companies within individual states [1]. Each state enters an exclusive WIC contract with a single formula company to serve that state's low‐income households with subsidized products [1]. As a result of these agreements, research suggests that supermarkets offer more preferential shelving to the WIC‐contract brand's infant formula, and pediatricians in each state may recommend the contract‐awarded formula brand over others [1]. Furthermore, according to research published by the USDA, the infant formula company that wins the WIC contract in a state has a significant market advantage in that state, with a monopoly on WIC sales and “spillover” effects extending into the non‐WIC market [1]. For example, Abbott, the formula company that owns the halted Michigan plant, has monopolies on WIC in almost two‐thirds of the country [5]. The federal government has requested temporary flexibility in exclusive contracts, thus allowing WIC vouchers to be used to purchase other brands of available formula [6]. However, this stopgap solution is unlikely to result in long‐term improvements in states’ use of single suppliers.

## A CLOSED‐OFF FORMULA MARKET

Lastly, the lack of imports of infant formula in the United States has substantially contributed to the current shortage [1]. This absence is attributed to the substantial 17.5% tariffs imposed on most imported infant formulas, and the strict nutritional guidelines set by the Food and Drug Administration (FDA] that make it practically impossible for formulas produced outside of the United States to be imported in [7]. Experts have criticized this “closed‐market policy” as denying safe infant formula from being sourced from Europe and Canada, instead primarily serving to protect the domestic monopoly from outside competition [1]. Furthermore, experts argue that the sole reliance of the United States on a domestic baby formula supply has resulted in increased vulnerability, whereas utilizing a variety of approved suppliers could have been protective against the current formula crisis [1]. The FDA subsequently issued new guidance that relaxes previous importation restrictions for 6 months to curb the infant formula shortage while maintaining safety checks [7]. However, this temporary flexibility in imports is a stopgap solution that will not improve long‐term resiliency against another domestic disruption in infant formula [7].

## CONSEQUENCES OF INFANT FORMULA SHORTAGES IN THE UNITED STATES

After examining the underlying causes of the US baby formula shortage, it is necessary to analyze the potential accompanying consequences. According to the CDC, an estimated one in every five babies receives baby formula within the first 2 days of life [8]. By 3 months, less than half are exclusively nursing, signaling that caregivers are supplementing with the baby formula [8]. While the widespread effects of baby formula shortages are evident, the consequences are not equally distributed. Research shows that shortages disproportionately affect rural and low‐income parents, especially as caregivers living in poverty are more likely to report supplementing with baby formula within a baby's first 3 months of life [8]. Furthermore, parents from racial and ethnic minorities are more likely to report using baby formula within these first 3 months compared to their White counterparts [8]. In addition to observed population‐level consequences, it is crucial to consider the individual consequences of baby formula substitutions, unhealthy eating behaviors, and early introduction to solids [8]. First, the creation of homemade baby formula by parents can be potentially dangerous for their children and may not meet their required nutritional needs [9].

Additionally, caregivers may water down infant formula to prolong its use, but this can result in poorer nutrition and problematic health outcomes for an infant [10]. Secondly, several maladaptive eating behaviors may arise in infants given formula shortages, such as through parents inadvertently encouraging their children to undereat due to rationing of baby formula, Conversely, children could also be encouraged to engage in overeating maladaptive behaviors, as caregivers that are concerned about underweight infants may subsequently pressure or force food intake [10]. Lastly, this shortage may result in an infant starting solid earlier than is developmentally appropriate [8]. Early introduction of solids poses concerns of choking, disruptions to the intestinal microbiome, unknown allergies, and an increased risk of childhood obesity [10]. The potential consequences caused by the infant formula shortage are evident for vulnerable caregivers, baby formula replacements, under‐ and overeating behaviors, and developmentally inappropriate introductions to solids, but more consequences will likely arise.

## WHERE DOES THE UNITED STATES GO FROM HERE?

Onlookers may argue caregivers should simply switch to another baby formula brand [11]. However, this solution is not always possible due to access issues, allergies, differing supplement needs, and the slow transition required to avoid gastrointestinal issues [11]. Others may argue that women should exclusively breastfeed their infants [12]. However, this can be challenging due to difficulty in getting the infant to latch, pain or discomfort, lack of social support, inability to pump in professional settings, lack of knowledge about the benefits of breastfeeding, outside pressure to use baby formula, and costs associated with items like breast pumps and storage bags [12]. As solutions surrounding switching baby formula and breastfeeding lack feasibility, other potential solutions should be explored to combat the baby formula shortage. We must focus on how these supply chains are impacting low and middle‐income countries. We can learn from this problem by preparing our emergency systems. We also must be better advocates to limit substitutes that encourage monopolies on medical necessity. For individual‐level solutions, experts recommend that caregivers temporarily supplement with whole cow's milk, obtain baby formula samples from pediatricians, purchase store‐brand baby formulas, and breastfeed if possible. However, these short‐lived solutions place the burden on caregivers and may be impractical given cost, allergies, nutritional needs, and other barriers. For structural‐level solutions to address current baby formula shortages, the United States has temporarily relaxed import restrictions [7] and requested flexibility in state WIC contracts [6]. However, these stopgaps fail to sustainably address the monopolized domestic system that caused this baby formula crisis. For the immediate future, individual‐level solutions are necessary for caregivers to meet their children's nutritional needs, but a multi‐level approach with lasting structural changes is necessary to prevent future infant baby formula shortages in the United States.

## AUTHOR CONTRIBUTIONS

Lila Chamlagai: Conceptulizations, formal analysis, Project adminstration, Resources, Writing‐Original draft, revewing and editing. Elizabeth Silva: Formal anlysis, writing original draft, revewing and editing. Ana Alikani: Conceptulization, formal analysis, resources, writing original draft, revewing and editing.

## CONFLICT OF INTEREST

Lila Chamlagai is an Editorial Board member of Public Health Challenges. and a co‐author of this article. To minimize bias, they were excluded from all editorial decision‐making related to the acceptance of this article for publication.

## FUNDING STATEMENT

There is no funding for the development of this paper.

## ETHICS STATEMENT

This is a Commentary piece. There is no need for ethical approval.

## Data Availability

Not applicable.
